# Influence of incorporating dried fruits on dairy drinks characteristics focusing on their antimicrobial effects

**DOI:** 10.5455/javar.2024.k747

**Published:** 2024-03-12

**Authors:** Neveen S. M. Soliman, Ayah B. Abdel-Salam, Shimaa R. Emam, Ahmed Orabi, Sara M. Nader, Mena Saad

**Affiliations:** 1 Food Hygiene and Control Department, Faculty of Veterinary Medicine, Cairo University, Giza, Egypt; 2 Pharmacology Department, Faculty of Veterinary Medicine, Cairo University, Giza, Egypt; 3 Microbiology Department, Faculty of Veterinary Medicine, Cairo University, Giza, Egypt; 4 Zoonoses Department, Faculty of Veterinary Medicine, Cairo University, Giza, Egypt

**Keywords:** Dairy drinks, dried fruits, sensory parameters, chemical parameters, *Pseudomonas aeruginosa*, *Bacillus cereus*

## Abstract

**Objective:**

The study was designed to show the effect of adding different levels of dried fruit extracts for 14 days on sensory and chemical parameters in dairy drinks. The survival of *Pseudomonas aeruginosa* and *Bacillus cereus* in artificially contaminated dairy drinks fortified with these extracts was also studied.

**Materials and Methods:**

The freshly watery extracts and nonaqueous extracts of dried fruits were prepared by rotary evaporators and solvents, respectively. The determination of the minimum inhibitory concentration of dried fruit extracts was achieved using the disc diffusion test. The sensory evaluation of samples was done, while the chemical parameters of the examined samples were determined by the calibrated analyzer. In addition, the degree of survival of *P. aeruginosa* and *B. cereus* in inoculated milk samples was also estimated.

**Results:**

In pasteurized and Rayeb milk samples, the water extract of carob and all alcoholic dried fruit extracts had a significant effect on compositional parameters in comparison to control samples. At day 14 of pasteurized milk storage, the watery (20.0%) and alcoholic (10.0%) extracts of carob significantly improved its sensory parameters.

**Conclusion:**

Based on the survival results, all utilized dried fruit extracts had a significant inhibitory effect on *P. aeruginosa* and *B. cereus* growth in the fortified milk samples at the end of storage. This trial of the survival of these new dairy drinks is the first investigation, particularly in the Middle East. Extracts of utilized dried fruits have prospective functions that enhance dairy drink characteristics.

## Introduction

By comparison between food items, the number of unique milk products on the commercial market has increased significantly everywhere. The new milk drinks fortified with dried fruits, especially pasteurized and Rayeb milk, are preferable because of their elevated nutritional characteristics, exhilarating effects, and flavor [1]. An attractive fermented milk product, Rayeb is processed by spontaneous milk fermentation. The microflora known as lactic acid bacteria (LB) has been shown to improve the quality of Rayeb milk, and some have antibacterial properties against bacteria such as *Staphylococcus aureus* (*S. aureus*), *Escherichia coli* (*E. coli*), and *Listeria monocytogenes* [2].

It is widely acknowledged that microbial contamination causes milk and dairy products to deteriorate, which is a serious problem. Although many inhibitory chemicals are hazardous, many types are used to stop these products from spoiling. Based on the method code (2009.01) by the Association of Official Analytical Chemists (AOAC), raisins contain 10.0% of the *Food and Drug Administration’s* daily recommended fiber [3,4] and represent a prebiotic origin in dairy drinks. Prebiotics enhance probiotic multiplication by giving sugar to LB [5].

Marčetić et al. [6] reported that ethyl alcohol prunes extract illustrated antibacterial actions against *S. aureus*, *E. coli*, and *Pseudomonas aeruginosa* strains. Moreover, the antibacterial properties of methyl alcohol prune extract have been confirmed against *E. coli*, *P. aeruginosa*, *Bacillus subtilis*, and *S. aureus*. In addition, the water extract of carob has inhibitory action against certain strains of *S. aureus*, *L. monocytogenes* (*L. monocytogenes*), *Bacillus cereus*, *B. subtilis*, *P. aeruginosa*, and *E. coli* [7].

Most previous investigations were very scanty on incorporating these dried fruits into dairy drinks, especially the antimicrobial effect, the degree of consumers’ acceptability with respect to this effect, and the influence of these dried fruits on the chemical parameters of dairy drinks. Therefore, this study aimed to pinpoint and evaluate the effect of extracts of dried fruits on the sensory and chemical parameters of pasteurized milk and Rayeb milk. In addition, the degree of antimicrobial effect of dried fruits at different concentrations during storage in a fridge (5°C) of fortified dairy products was evaluated.

## Materials and Methods

### Dried fruits used

The four dried fruit samples were purchased from the “Harraz for Food Industry and Natural Products” in Cairo, Egypt, and then kept at 5°C until utilized. Dried fruit samples were thoroughly washed with distilled water, then cut into small pieces, and finally dried in a hot air oven (Blender, GmbH, Germany) at 40°C (to keep the fruits energetic substances) for three successive days or until they reached a constant weight [8]. After that, the fine powder was made from the dried fruit samples using a blade mixer, SPEX-SamplePrep-GenoGrinder^®^ (Metuchen, USA), and the powder samples were stored at −20°C for further use.

### Freshly watery extract preparation of dried fruits

The dry fruit watery extracts were obtained according to the method of Twarogowska et al. [9]. Exactly 200 gm of dried powdered fruit was soaked in 2,000 ml of distilled water at 70°C for 1 h in a water bath (Memmert, Germany) and then strained through cheesecloth. In a rotary evaporator operating at 60°C under pressure (760 mmHg), the filtrate was concentrated (100.0%). The resulting water extracts were transferred to 500-ml glass bottles, autoclaved for 15 min at 121°C, and then kept chilled at 4°C until use in a refrigerator (Toshiba, Nu-frost).

### Nonaqueous solvent extract of dried fruit preparation

Ethanol and methanol extracts from dry fruit were obtained according to the methods of Ali et al. [10] and Khamis et al. [11], respectively. The solvents used to extract each dried powdered fruit were methanol 80.0% for the fig and prunes and ethanol 80.0% for the raisins and carob. This extraction process took at least 24 h, and the solvents were then percolated 5–7 times until completely exhausted. According to the procedure of Emam et al. [12], these extracts were subsequently filtered using Whatman No. 1 filter paper and condensed under pressure (760 mmHg) using a rotary evaporator (Heidolph 2000, Germany) at a temperature not more than 40°C. After being collected, the semisolid extract was dried for 24 h at 37°C in an incubator. The extracted materials were weighed and kept chilled at 5°C until needed (concentration 100.0%). Analytical-grade methanol and ethanol were purchased from El-Gomhoryia Company, Cairo, Egypt.

### Milk preparation

The raw cow’s milk was supplied by the Faculty of Agriculture, Cairo University. Milk was heated at 85°C for 10 min and then cooled to 40°C. The chemical components of milk were analyzed based on AOAC [13]. The Rayeb milk was processed from heated cow milk in a water bath (BaneBio, Frederick, MD) at 85°C for 10 min and then cooled to 40°C. The starter culture utilized for Rayeb processing was prepared according to manufacturer instructions in hygienic surroundings. The previous heat-treated milk was inoculated with a 0.1 gm/l milk proportion of starter culture (ABT-5 *Streptococcus thermophilus*, *Lactobacillus acidophilus*, and *Bifidobacterium lactis* Bb12, Chr. Hansen’s company, Denmark, catalog # TH-4^®^, LA-5^®^, UABla-12^™^), incubated for 5 h, kept at 4°C for 12 h, then mixed for 5 min. After that, the samples of Rayeb milk were preserved in a sterilized container at 5°C for 14 days.

Each type of milk sample with four extracts of dried fruits was examined at zero time and after 3, 6, 9, 12, and 14 days of storage, based on Abou-Dobara et al. [14]. It was divided into nine portions for water extracts (20.0%) of fig, raisins, prunes, and carob and alcoholic extracts of fig (20.0%), raisins (10.0%), prunes (20.0%), and carob (10.0%), and a control without any extracts of fruits for sensory and chemical examination.

### Determination of the minimum inhibitory concentration (MIC) of dried fruit extracts

The disc diffusion test was used to evaluate the MIC of dried fruit water and alcoholic extracts using a paper disk, which was located on top of an inoculated agar plate soaked with 20 µl of each extract, and their efficiency in controlling tested bacteria was evaluated. Using slipping calipers and the National Committee for Clinical Laboratory Standards, the diameters of the inhibition zones (three times) were measured [15]. The microorganisms used were *S. aureus* ATCC 43300, *P. aeruginosa* ATCC 27853, *L. monocytogenes* ATCC 19115, *B. cereus* ATCC 33018, and *E. coli* ATCC 25922, obtained from the Department of Microbiology, Faculty of Veterinary Medicine, Cairo University.

### Sensory examination of the milk samples

Each pasteurized and Rayeb milk sample was numbered and offered in a 75-ml plastic container to three panelists from the staff of the Food Hygiene department, Cairo University, when fresh and after 3, 6, 9, 12, and 14 days using a 20-point system. Ultimately, the outputs of this evaluation were documented in a particular scorecard based on Clark et al. [16].

### Chemical parameters examination of the milk samples

All determined values were achieved in triplicate. A calibrated analyzer (4 lines × 16 characters, 240 Volt-1.6 ampere max., Bulgaria) was used to measure chemical aspects of the examined milk samples, including total protein, solids nonfat (SNF), salt, and sugar percentages. The fat percentage was measured by the Gerber method as documented by International Organization for Standardization (ISO) [17,18]

### Study of P. aeruginosa and B. cereus survival in the examined milk samples

From the results of the MIC, *P. aeruginosa* and *B. cereus* were chosen for further procedures. Bacterial strains were preserved at 5°C until use. For refreshment, 5 ml of brain heart broth (CM1135, Oxoid) was inoculated with each bacterial strain and incubated at 37°C for 24 h. After that, the cells were pelleted by centrifugation (Thermo-Fisher, USA) at 3,500 RPM/10 min. The washing of the pellet three times was achieved with 0.1% peptone water (M028, Himedia). Re-suspension of cells was achieved with the same diluent. For count determination in the suspension, a 10-fold serial dilution was achieved, and the cultivation of bacterial strains from these dilutions was done by spreading technique (0.1 ml inoculum) to find an ultimate concentration of cells of 10 log CFU/ml suspension.

Each pasteurized and Rayeb milk was divided into nine portions for water extracts (20%) of fig, raisins, prunes, and carob and alcoholic extracts of fig (20.0%), raisins (10.0%), prunes (20.0%), and carob (10.0%), and milk without any extracts. For each type of dried fruit and each concentration, milk was inoculated with approximately 8.95 and 8.69 log CFU of *P. aeruginosa* and *B. cereus*, respectively. The milk samples were inoculated by strains of bacteria to ensure the survival of *P. aeruginosa* and *B. cereus* over 14 days of refrigeration, based on Dumalisile et al. [19]. The trial was done in triplicates, and the average concentrations of both bacteria were reported.

### Preparation of milk samples and enumeration of inoculated bacteria

As a diluent, 9 ml of 0.1% peptone water (M028, Himedia) was aseptically exchanged with 1 ml of each type of milk sample. The first dilution was blended for 10 sec by Stomacher (Seward, Worthing, UK) to get a 1/10 dilution. Then, 1 ml of the first dilution was relocated into 9 ml of diluents to obtain decimal dilutions based on ISO [17,18] and 0.1 ml of these dilutions were spread on plates of agar. Duplicated plates of mannitol egg yolk polymyxin agar (Himdia, M 636S) were used for the plating of *B. cereus*, and duplicated plates of *Pseudomonas* agar media (Himdia, M085) were used for *P. aeruginosa*, following Adam et al. [20].

### Statistical analysis of data

The one-way analysis of variance was used to estimate the *p*-value (*p* ≤ 0.05) for significant differences between the mean numbers of the determined items for various types and concentrations of dried fruit extracts in pasteurized and Rayeb milk samples using *post-hoc* and Tukey HSD (Honestly Significant Difference) through SPSS (Statistical Package for the Social Sciences) version 25.

## Results and Discussion

In Tables 1 and 2, the antibacterial effect of raisins water extract appeared starting from a concentration of 10.0% and beginning at 5.0% in an alcoholic extract of raisins and carob. For that reason, the concentrations were elevated to 10.0%, 20.0%, and 30.0%. The minimum concentration of water extract with an antibacterial effect was 20.0% for all fruits used, but raisins were still the most effective. This may be attributed to elevated percentages of phenolic components and direct exposure of the bacteria to these components; all these synergistic factors contribute to increasing the antibacterial effects of raisins. The use of dried fruits is safe and does not share in foodborne diseases due to a combination of inhibitor factors and the stopping of watering before the collection of dry fruit [21].

The minimum effective concentration of the alcoholic extract was 10.0% for raisins and carob and 20.0% for fig and prunes, but raisins showed the highest antibacterial effect. The alcoholic extract had an effect at a high concentration on nearly all the tested bacterial strains, but the best effect was still on the selected strains. Therefore, it was chosen to complete the experimental study with a minimum concentration of the fruit extracts. These differences in the minimum effective concentration of dried fruit extracts against the bacterial strains may be attributed to the type and concentration of extract prepared from each fruit [22].

**Table 1.** MIC of watery extract of used dried fruits.

Tested strains	Inhibition zone diameter (mm)																			
	FW concentration (%)					RW concentration (%)					PW concentration (%)					CW concentration (%)				
	2.5	5	10	20	30	2.5	5	10	20	30	2.5	5	10	20	30	2.5	5	10	20	30
*S. aureus*	0	0	1.3	7.3	13	0	0	4	9	15	0	0	0	4	9	0	0	1	3.3	9
*P. aeruginosa*	0	0	5	11	19.3	0	0	7	13	22	0	0	2	9.3	14	0	0	2	11	18
*L. monocytogenes*	0	0	5	9	13	0	0	9	13	17.3	0	0	1	5	11.3	0	0	2.3	9	16.3
*B. cereus*	0	0	7	12	17.3	0	0	9.3	15	24	0	0	2	9	16.3	0	0	3	13.3	21
*E. coli*	0	0	4	9	14	0	0	6	11	16.3	0	0	0	3	7	0	0	0	5	11

Values an average of triplicates, values equal mm, FW = fig water extract, RW = raisin water extract, PW = prunes water extract, CW = carob water extract, FM = fig methanol extract, RE = raisin ethanol extract, PM = prunes methanol extract, CE = carob ethanol extract.

**Table 2.** MIC of alcoholic extract of used dried fruits.

Tested strains	Inhibition zone diameter (mm)																			
	FM concentration (%)					RE concentration (%)					PM concentration (%)					CE concentration (%)				
	2.5	5	10	20	30	2.5	5	10	20	30	2.5	5	10	20	30	2.5	5	10	20	30
*S. aureus*	0	0	5	8.7	13	0	3	9.3	13.3	22	0	0	5	7.7	11	0	6	7.7	12	14
*P. aeruginosa*	0	1	9	19.3	22.3	0	7.3	17	21	24	0	4.3	8.3	16	19	0	7	11.3	17	21.3
*L. monocytogenes*	0	1	7	18	23.7	0	5.7	14	19.3	21	0	4	9	13.7	16.7	0	6.3	9.7	13.3	16.3
*B. cereus*	0	3	9	21.3	27	0	9.3	21.3	24.7	27	0	5.7	11.3	19.7	22	0	9	22.3	27	32
*E. coli*	0	2	6	17	20	0	6	11.7	16.3	22	0	2	7	10.3	17	0	9.3	11	15.7	22

Values an average of triplicates, values equal mm.

In this study, the sensory trial was done only at the selected concentration (MIC) to determine the degree of acceptability for the consumer of fortified products with different fruit flavors. The alcoholic extract was better in sensory quality and attractiveness than the water extract, which was less preferred by the panelists for each dried fruit. This may be attributed to the concentration of the active principles in the alcoholic extract (more powerful extraction by alcohol) than the water extract [23].

In Table 3, at day 14 of pasteurized milk sample storage, the water extracts of raisin and carob and the alcoholic extracts of prunes and carob had a significant enhancing effect compared to the control samples. In Rayeb milk samples, all extracts had a nonsignificant (*p* > 0.05) effect on their sensory parameters but were still accepted by panelists. This was in agreement with the results of previous studies [1,3,24]. The samples of Rayeb had a more balanced taste and were more accepted than the same concentrations in pasteurized milk samples; this agreed with the study results of Tami et al. [25]. Based on a significant output of the Debbabi et al. [26] study, the panelists’ acceptability evaluation was that 52.0% agreed to raw milk Rayeb, but it was processed from pasteurized milk with 24.0%.

According to the chemical analysis of pasteurized and Rayeb milk, the carob water extract and all alcoholic extracts had a significant effect in comparison to samples without dried fruit extract for fat%, protein%, sugar%, SNF%, and salt% (Tables 4 and 5). Based on the previous study by Srour et al. [24], they found an elevation in the protein percentage, consequently elevating the milk compositional value.

**Figure 1.**
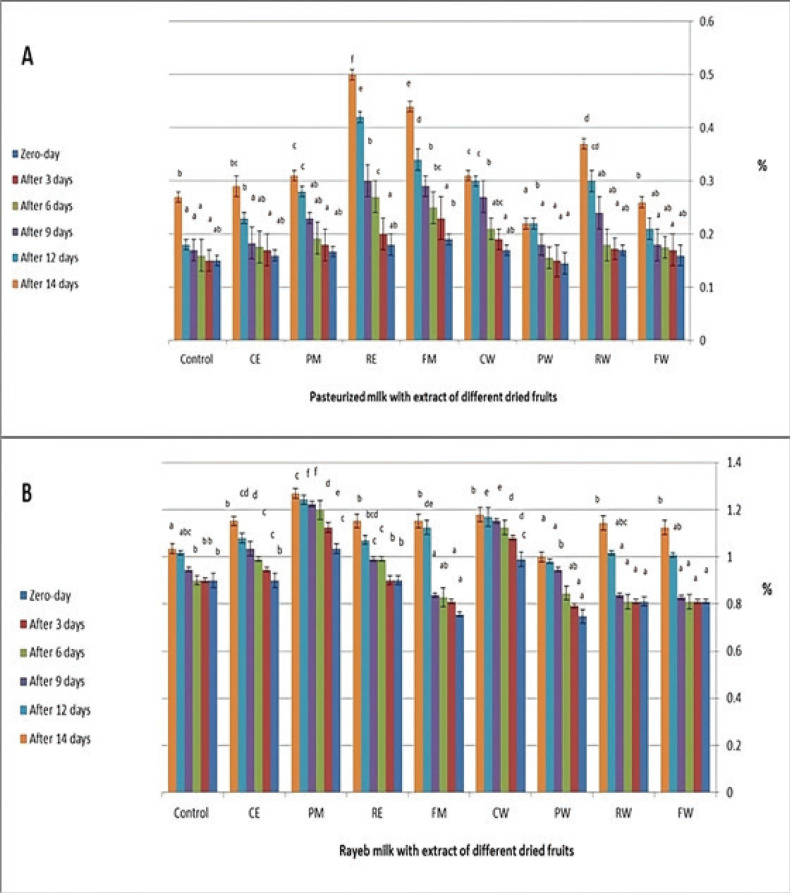
(A) Changes in titratable acidity % during storage periods for the examined pasteurized milk samples fortified with dried fruits. (B) Changes in titratable acidity % during storage periods for the examined Rayeb milk samples fortified with dried fruits. The values are sitting as mean and standard deviation bar for ± the standard deviation, for each storage period: a significant (*p* < 0.05) difference only between different letters above each column.

The water extracts of raisins, carob, and alcoholic extracts of fig, raisins, and prunes had a significant effect on the titratable acidity percentage in all samples at the end of storage (Fig. 1A and B). The increasing milk acidity may be referred to as sugar fermentation, which increases in alcoholic extracts of used dried fruits due to high sugar concentrations [1].

**Figure 2.**
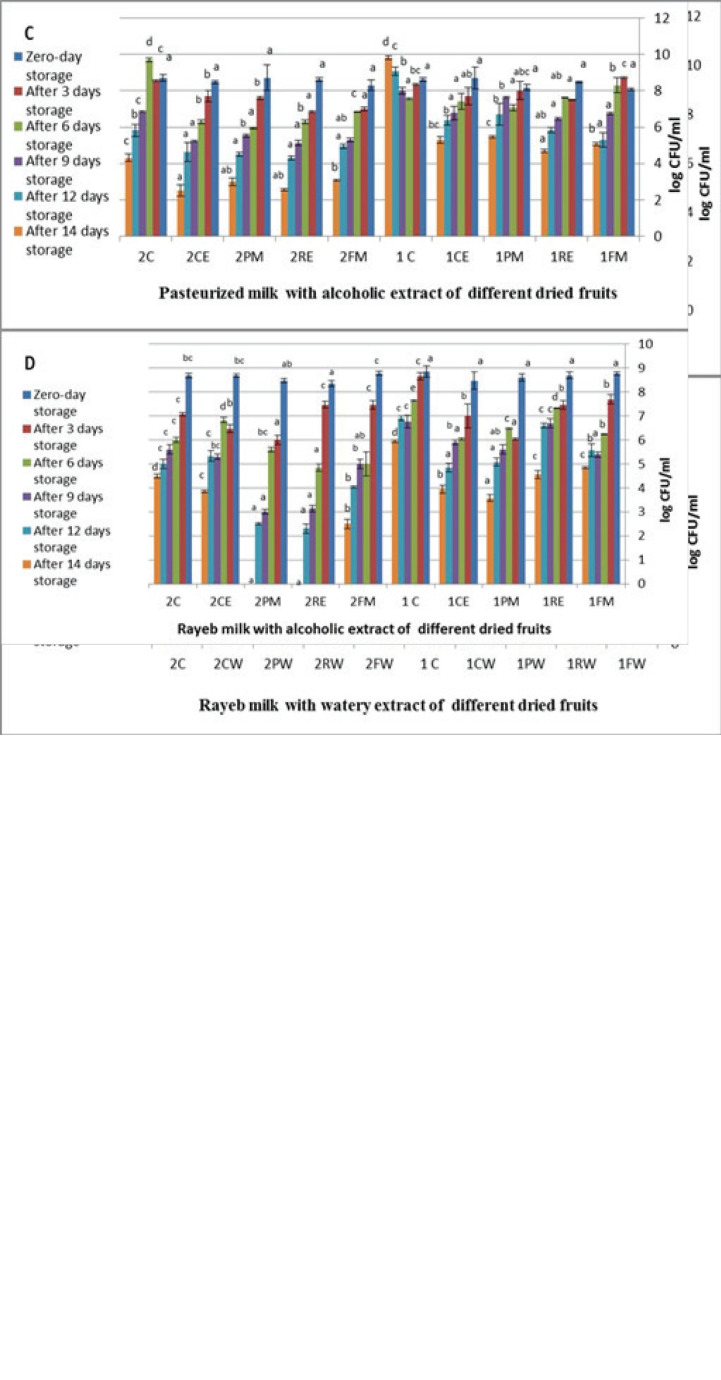
(A) Log reduction of inoculated *P. aeruginosa* and *B. cereus* in the pasteurized milk samples with watery extract of dried fruits at different storage periods. (B) Log reduction of inoculated *P. aeruginosa* and *B. cereus* in the Rayeb milk samples with watery extract of dried fruits at different storage periods. (C) Log reduction of inoculated *P. aeruginosa* and *B. cereus* in the pasteurized milk samples with alcohol extract of dried fruits at different storage periods. (D) Log reduction of inoculated *P. aeruginosa* and *B. cereus* in the Rayeb milk samples with alcohol extract of dried fruits at different storage periods. The values are sitting as mean and standard deviation bar for ± the standard deviation. 1 = *P. aeruginosa*, 2 = *B. cereus*, C = Control. FM = fig methanol extract, RE = raisin ethanol extract, PM = prunes methanol extract, CE = carob ethanol extract, for each storage period and each bacterial strain: a significant (*p* < 0.05) difference only between different letters above each column.

According to the *P. aeruginosa* and *B. cereus* survival results (Fig. 2), all water and alcoholic extracts of utilized dried fruits had a significant inhibitory effect on *P. aeruginosa* and *B. cereus* growth in the pasteurized and Rayeb milk samples at the end of the storage period. This log reduction may be attributed to the acid output of dried fruit extracts. Also, the starter bacteria’s presence may be an additional suppression of Rayeb milk spoilage [27]. It is also an added value to produce new fortified milk products that are acceptable to consumers at suitable prices. The extract of dried fruits needs wide verification to ensure its level of antimicrobial influence in such products.

**Table 3.** Grading of the examined pasteurized milk and Rayeb milk based on sensory aspects at different storage periods.

Period	Sample	Flavor (10)		Body and texture (5)		Appearance (5)		Total score (20)	
		A	B	A	B	A	B	A	B
Zero-day storage	FW	7	8	4	5	4	5	15 ± 1.0^abc^	18 ± 1.0^ab^
	RW	7	8	4	5	3	4	14 ± 1.0^ab^	17 ± 1.0^ab^
	PW	8	9	5	5	3	3	16 ± 1.0^bcd^	17 ± 1.0^ab^
	CW	9	7	5	5	4	4	18 ± 1.0^de^	16 ± 1.0^a^
	FM	6	7	4	5	4	5	14 ± 1.0^ab^	17 ± 1.0^ab^
	RE	6	8	4	5	3	5	13 ± 1.0^a^	18 ± 1.0^ab^
	PM	9	9	5	5	3	4	17 ± 1.0^cde^	18 ± 1.0^ab^
	CE	9	9	5	5	4	5	18 ± 1.0^de^	19 ± 1.0^b^
	Control	10	9	5	5	5	5	20 ± 0.577^e^	19 ± 1.0^b^
After 3 days storage	FW	6	8	4	5	4	5	14 ± 1.0^ab^	18 ± 1.0^ab^
	RW	7	8	4	5	4	4	15 ± 1.0^abc^	17 ± 1.0^ab^
	PW	8	9	5	5	3	3	16 ± 1.0^bcd^	17 ± 1.0^ab^
	CW	9	7	5	5	4	4	18 ± 1.0^de^	16 ± 1.0^a^
	FM	6	7	4	5	4	5	14 ± 1.0^ab^	17 ± 1.0^ab^
	RE	6	8	4	5	3	5	13 ± 1.0^a^	18 ± 1.0^ab^
	PM	9	9	5	5	3	4	17 ± 1.0^cde^	18 ± 1.0^ab^
	CE	10	9	5	5	4	5	19 ± 1.0^e^	19 ± 1.0^b^
	Control	10	9	5	5	5	5	20 ± 0.577^e^	19 ± 1.0^b^
After 6 days storage	FW	6	8	4	5	4	5	14 ± 1.0^ab^	18 ± 1.0^bc^
	RW	7	8	4	5	4	4	15 ± 1.0^abc^	17 ± 1.0^abc^
	PW	8	9	5	5	3	3	16 ± 1.0^bcd^	17 ± 1.0^abc^
	CW	9	6	5	5	4	4	18 ± 1.0^de^	15 ± 1.0^a^
	FM	6	7	4	5	4	3	14 ± 1.0^ab^	15 ± 1.0^a^
	RE	6	8	4	5	3	5	13 ± 1.0^a^	18 ± 1.0^bc^
	PM	9	9	5	5	3	3	17 ± 1.0^cde^	17 ± 1.0^abc^
	CE	10	7	5	5	4	4	19 ± 1.0^e^	16 ± 1.0^ab^
	Control	10	9	5	5	5	5	20 ± 0.577^e^	19 ± 1.0^c^
After 9 days storage	FW	6	8	3	5	4	5	13 ± 1.0^ab^	18 ± 1.0^bc^
	RW	7	8	4	5	4	4	15 ± 1.0^bcd^	17 ± 1.0^abc^
	PW	8	9	5	5	3	3	16 ± 1.0^cde^	17 ± 1.0^abc^
	CW	8	6	5	5	4	4	17 ± 1.0^de^	15 ± 1.0^a^
	FM	6	7	4	5	4	3	14 ± 1.0^abc^	15 ± 1.0^a^
	RE	6	8	3	5	3	5	12 ± 1.0^a^	18 ± 1.0^bc^
	PM	7	9	5	5	3	3	15 ± 1.0^bcd^	17 ± 1.0^abc^
	CE	9	7	4	5	4	4	17 ± 1.0^de^	16 ± 1.0^ab^
	Control	9	9	4	5	5	5	18 ± 1.0^e^	19 ± 1.0^c^
After 12 days storage	FW	5	7	3	5	4	4	12 ± 1.0^ab^	17 ± 1.0^bc^
	RW	5	8	3	5	4	4	12 ± 1.0^ab^	17 ± 1.0^bc^
	PW	6	9	4	4	3	3	13 ± 1.0^ab^	16 ± 1.0^bc^
	CW	6	6	4	5	4	4	14 ± 1.0^bc^	15 ± 1.0^ab^
	FM	6	6	3	4	4	3	13 ± 1.0^ab^	13 ± 1.0^a^
	RE	5	8	3	5	3	5	11 ± 1.0^a^	18 ± 1.0^c^
	PM	7	8	3	5	3	3	13 ± 1.0^ab^	16 ± 1.0^bc^
	CE	8	7	4	4	4	4	16 ± 1.0^c^	15 ± 1.0^ab^
	Control	8	8	3	5	3	4	14 ± 1.0^bc^	17 ± 1.0^bc^
After 14 days storage	FW	3	7	3	4	4	4	10 ± 1.0^ab^	15 ± 1.0^ab^
	RW	5	8	3	4	4	4	12 ± 1.0^bc^	16 ± 1.0^b^
	PW	4	9	4	4	3	3	11 ± 1.0^ab^	16 ± 1.0^b^
	CW	5	6	3	5	4	4	12 ± 1.0^bc^	15 ± 1.0^ab^
	FM	4	6	3	4	3	3	10 ± 1.0^ab^	13 ± 1.0^a^
	RE	3	7	3	4	3	4	9 ± 1.0^a^	15 ± 1.0^ab^
	PM	6	6	3	4	3	3	12 ± 1.0^bc^	13 ± 1.0^a^
	CE	6	7	4	3	4	3	14 ± 1.0^c^	13 ± 1.0^a^
	Control	5	7	1	4	3	4	9 ± 1.0^a^	15 ± 1.0^ab^

A = pasteurized milk, B = Rayeb milk, FW = fig water extract, RW = raisin water extract, PW = prunes water extract, CW = carob water extract, FM = fig methanol extract, RE = raisin ethanol extract, PM = prunes methanol extract, CE = carob ethanol extract. ± = standard deviation, for each storage period: all different letters significant (*p* < 0.05) with each other in the same column, all the same letters were nonsignificant (*p* > 0.05) difference with each other in the same column.

**Table 4.** Chemical parameters changes of pasteurized milk after addition of extracts of dried fruits.

Sample		Fat %	Protein %	Sugars %	SNF %	Salt %
Pasteurized milk without extracts addition		3.35 ± 0.10^bc^	2.68 ± 0.01^a^	3.80 ± 0.10^a^	7.18 ± 0.01^a^	0.67 ± 0. 01^a^
Fortified pasteurized milk with different fruit extracts (at zero-day of storage)	FW 20.0%	3.56 ± 0.20^c^	4.01 ± 0.01^d^	4.98 ± 0.01^b^	9.49 ± 0.01^b^	1.80 ± 0.01^e^
	RW 20.0%	3.46 ± 0.10^bc^	3.99 ± 0.01^d^	5.31 ± 0.01^c^	10.12 ± 0.01^d^	1.08 ± 0.01^d^
	PW 20.0%	3.21 ± 0.10^ab^	3.89 ± 0.01^c^	5.55 ± 0.01^d^	10.47 ± 0.01^e^	0.97 ± 0.01^c^
	CW 20.0%	2.94 ± 0.02^a^	3.55 ± 0.02^b^	5.07 ± 0.02^b^	9.56 ± 0.01^c^	0.88 ± 0.01^b^
	FM 20.0%	5.17 ± 0.10^e^	7.68 ± 0.01^e^	10.98 ± 0.01^e^	20.68 ± 0.01^f^	1.91 ± 0.02^f^
	RE 10.0%	6.43 ± 0.1^f^	10.43 ± 0.01^h^	14.91 ± 0.01^h^	28.07 ± 0.02^i^	2.59 ± 0.03^h^
	PM 20.0%	3.94 ± 0.03^d^	8.46 ± 0.02^f^	12.11 ± 0.01^f^	22.79 ± 0.02^g^	2.09 ± 0.01^g^
	CE 10.0%	4.99 ± 0.035^e^	9.50 ± 0.03^g^	12.97 ± 0.01^g^	25.74 ± 0.01^h^	2.10 ± 0.01^g^

FW = fig water extract, RW = raisin water extract, PW = prunes water extract, CW = carob water extract, FM = fig methanol extract, RE = raisin ethanol extract, PM = prunes methanol extract, CE = carob ethanol extract. ± = standard deviation, for fat%, protein, sugar, SNF, and salts%: all different letters significant (*p* < 0.05) with each other in the same column, all the same letters were nonsignificant (*p* > 0.05) difference with each other in the same column.

**Table 5.** Chemical parameters changes of Rayeb milk after the addition of extracts of dried fruits.

Sample		Fat %	Protein %	Sugars %	SNF %	Salt %
Rayeb milk without extracts		3.2 ± 0.10^a^	3.0 ± 0.01^a^	4.20 ± 0.10^a^	8.20 ± 0.10^a^	0.79 ± 0.10^a^
Fortified Rayeb milk with different fruit extracts (at zero-day of storage)	FW 20.0%	4.06 ± 0.01^c^	4.6 ± 0.01^b^	5.48 ± 0.01^b^	10.51 ± 0.10^b^	1.92 ± 0.02^c^
	RW 20.0%	3.55 ± 0.01^b^	4.95 ± 0.01^c^	5.81 ± 0.01^c^	11.14 ± 0.01^cd^	1.2 ± 0.10^b^
	PW 20.0%	3.42 ± 0.01^b^	4.49 ± 0.01^b^	5.59 ± 0.01^b^	11.5 ± 0.50^d^	1.09 ± 0.01^b^
	CW 20.0%	3.54 ± 0.01^b^	4.77 ± 0.01^bc^	5.95 ± 0.01^d^	10.72 ± 0.02^bc^	1.0 ± 0.061^b^
	FM 20.0%	7.56 ± 0.01^g^	8.28 ± 0.01^d^	11.39 ± 0.07^e^	21.7 ± 0.01^e^	2.05 ± 0.05^cd^
	RE 10.0%	6.2 ± 0.1^f^	11.33 ± 0.33^g^	15.64 ± 0.02^h^	29.09 ± 0.01^h^	2.72 ± 0.10^e^
	PM 20.0%	4.63 ± 0.01^d^	9.06 ± 0.06^e^	12.82 ± 0.02^f^	23.81 ± 0.10^f^	2.23 ± 0.10^d^
	CE 10.0%	5.89 ± 0.01^e^	10.1 ± 0.01^f^	13.89 ± 0.01^g^	26.76 ± 0.10^g^	2.22 ± 0.10^d^

± = standard deviation, for fat%, protein, sugar, SNF, and salts%: all different letters only significant (*p* < 0.05) with each other in the same column.

## Conclusion

The water extracts (20.0%) of utilized dried fruits and alcoholic extracts (fig and prunes 20.0% and raisins and carob 10.0%) can be efficiently utilized to produce pasteurized and Rayeb milk with superior compositional values. The water extracts of raisin and carob and the alcoholic extracts of prunes and carob have to significantly ameliorate the sensory parameters of the pasteurized milk at the end of the storage period. Both extracts of all utilized dried fruits had a significantly decreasing impact on *B. cereus* and *P. aeruginosa* counts in pasteurized and Rayeb milk at the end of storage. Pasteurized and Rayeb milk manufacturers should be informed of these results to initiate the addition of these added-value extracts of dried fruits, which are friendly environmental additives, during the processing protocol of these creative modern fortified kinds of dairy products.

## List of abbreviations

AOAC, Association of Official Agricultural Chemists; ATCC, American type culture collection; h, hour; ISO, International Organization for Standardization; min, minute; mm, millimeter; mmHg, millimeter of mercury; RPM, revolution per minute.
